# CMV and Immunosenescence: from basics to clinics

**DOI:** 10.1186/1742-4933-9-23

**Published:** 2012-10-31

**Authors:** Rafael Solana, Raquel Tarazona, Allison E Aiello, Arne N Akbar, Victor Appay, Mark Beswick, Jos A Bosch, Carmen Campos, Sara Cantisán, Luka Cicin-Sain, Evelyna Derhovanessian, Sara Ferrando-Martínez, Daniela Frasca, Tamas Fulöp, Sheila Govind, Beatrix Grubeck-Loebenstein, Ann Hill, Mikko Hurme, Florian Kern, Anis Larbi, Miguel López-Botet, Andrea B Maier, Janet E McElhaney, Paul Moss, Elissaveta Naumova, Janko Nikolich-Zugich, Alejandra Pera, Jerrald L Rector, Natalie Riddell, Beatriz Sanchez-Correa, Paolo Sansoni, Delphine Sauce, Rene van Lier, George C Wang, Mark R Wills, Maciej Zieliński, Graham Pawelec

**Affiliations:** 1 Immunology Unit, Instituto Maimónides de Investigación Biomédica de Córdoba (IMIBIC)-Reina Sofia University Hospital-University of Cordoba, Cordoba, Spain; 2 Immunology Unit, Department of Physiology, University of Extremadura, Caceres, Spain; 3 University of Michigan, Department of Epidemiology, Center for Social Epidemiology and Population Health, Ann Arbor, Michigan, USA; 4 Division of Infection and Immunity, University College London, London, UK; 5 Infections and Immunity, Institut National de la Santé et de la Recherche Médicale (INSERM), Paris, France; 6 School of Cancer Sciences, University of Birmingham, Birmingham, UK; 7 University of Amsterdam, Amsterdam, The Netherlands; 8 Department of Vaccinology and Applied Microbiology, Helmholtz Centre for Infection Research, Braunschweig, Germany; 9 Department of Internal Medicine II, Center for Medical Research, University of Tübingen, Tübingen, Germany; 10 Laboratory of Molecular Immune-Biology, Hospital General Universitario Gregorio Marañón, Madrid and Laboratory of Immunovirology; Infectious Diseases Service, IBiS, Seville, Spain; 11 Department of Microbiology and Immunology, University of Miami Miller School of Medicine, Miami, Florida, USA; 12 Research Center on Aging, Sherbrooke, Canada; 13 Regenerative Medicine Group, Cranfield Health, Cranfield University, Cranfield, UK; 14 Institute for Biomedical Aging Research, University of Innsbruck, Innsbruck, Austria; 15 Department of Molecular Microbiology and Immunology, Oregon Health and Science University, Portland, Oregon, USA; 16 University of Tampere, Medical School, Tampere, Finland; 17 Brighton and Sussex Medical School, Brighton, UK; 18 Singapore Immunology Network, Singapore, Singapore; 19 IMIM (Hospital del Mar Research Institute), Univ. Pompeu Fabra, Barcelona, Spain; 20 Department of Gerontology and Geriatrics, Leiden University Medical Center, Leiden, The Netherlands; 21 University of British Columbia, Vancouver, Canada; 22 Department of Clinical Immunology, University Hospital Alexandrovska, Sofia, Bulgaria; 23 Department of Immunobiology and the Arizona Center on Aging, University of Arizona College of Medicine, Tucson, Arizona, USA; 24 University of Birmingham, Birmingham, UK; 25 Department of Internal Medicine and Biomedical Sciences, University of Parma, Parma, Italy; 26 Experimental Immunology, Academic Medical Center, Amsterdam, The Netherlands; 27 Division of Geriatric Medicine and Gerontology, Biology of Healthy Aging Program, Johns Hopkins University School of Medicine, Baltimore, Maryland, USA; 28 University of Cambridge Department of Medicine Addenbrookes Hospital, Cambridge, UK; 29 Department of Clinical Immunology and Transplantology, Medical University of Gdansk, Gdansk, Poland; 30 Mannheim Institute of Public Health, Social and Preventive Medicine (MIPH), University of Heidelberg, Mannheim Medical Faculty, Mannheim, Germany

## Abstract

Alone among herpesviruses, persistent Cytomegalovirus (CMV) markedly alters the numbers and proportions of peripheral immune cells in infected-vs-uninfected people. Because the rate of CMV infection increases with age in most countries, it has been suggested that it drives or at least exacerbates “immunosenescence”. This contention remains controversial and was the primary subject of the Third International Workshop on CMV & Immunosenescence which was held in Cordoba, Spain, 15-16^th^ March, 2012. Discussions focused on several main themes including the effects of CMV on adaptive immunity and immunosenescence, characterization of CMV-specific T cells, impact of CMV infection and ageing on innate immunity, and finally, most important, the clinical implications of immunosenescence and CMV infection. Here we summarize the major findings of this workshop.

## Introduction

**Figure 1:**
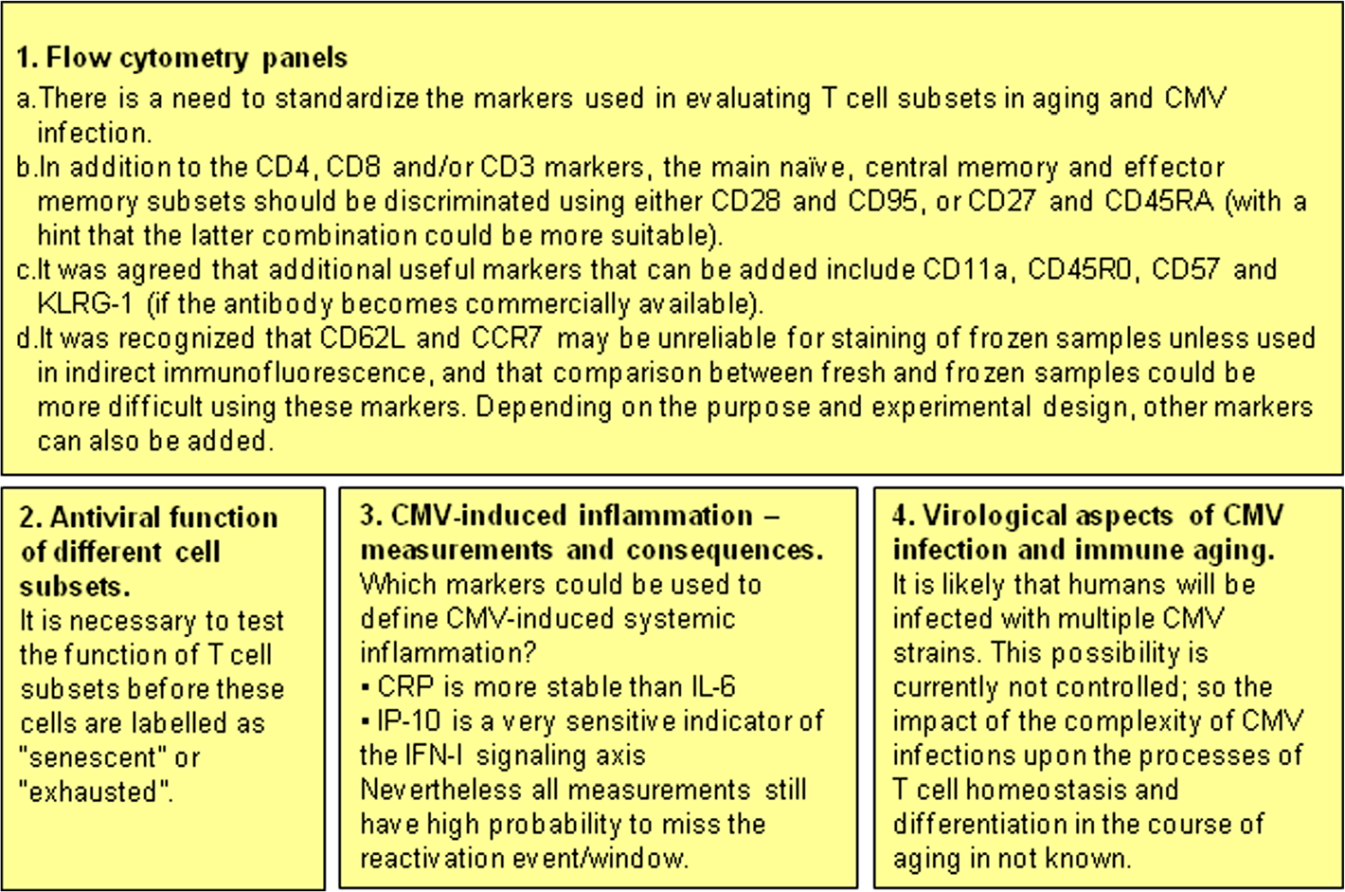
**CMV and immunosenescence: Open questions.** The relevance of the following questions on the role of CMV infection on immunosenescence and inflamm-aging were highlighted: **1**) the need to standardize the panel of mAbs used to asses lymphocyte subsets alterations, **2**) the role of each lymphoid subset in anti-CMV response, **3**) the significance of CMV-induced inflammation and **4**) the complexity of CMV infection in humans.

The impact of cytomegalovirus on the immune system and its relevance for the decline of immune function with ageing was discussed by international experts during the Third International Workshop on CMV & Immunosenescence held in Cordoba, Spain, 15-16^th^ March, 2012 (local organizer, Prof. R. Solana). This followed two previous Workshops held in Tubingen, Germany in 2009, and Cambridge, UK in 2010, the outcomes of which were summarized in this Journal by Pawelec et al. [1] and Wills et al. [2]. This commentary summarizes the major issues discussed at the Third Workshop in this series with an emphasis on those questions raised in the previous meetings that were left open. The meeting ended with a session of perspectives and closing remarks that included a discussion summary and several action items (Figure 1), and will be followed by a 4^th^ Workshop to be organized by Prof. P. Sansoni in Parma, Italy, 25-27^th^ March, 2013.

### Biomarkers of Immunosenescence and CMV infection

Pawelec (Tübingen, Germany) reviewed the recent advances in immunosenescence defined as the deleterious age-associated changes to immunity observed in all mammals studied so far. It was suggested that a better designation might be immune frailty as a continuous variable rather than a discrete state. The clinical impact of the observed age-associated changes in components of innate and adaptive immunity is mostly not clear in humans, and controversial data exist regarding the mechanisms of immunosenescence and the identification of new markers. In longitudinal studies of Swedish octogenarians and nonagenarians (OCTO and NONA) an immune risk profile (IRP) was proposed that was associated with increased mortality. The IRP was present in approximately 15% of individuals at baseline and 4 year mortality was almost double in individuals with IRP compared with non-IRP individuals. The main limitations of the IRP were emphasized. Thus, it has been shown to be relevant only in a small sample of very elderly people in one particular country but we do not know if these results can be extrapolated to larger and younger samples in different countries. The initial IRP analysis lacked the sophistication of modern immune analysis and it is unknown at which age it may become relevant and its association with underlying diseases is unclear. In addition, the IRP does not take into account many other clinical factors, such as nutritional status, psychological stress or inflammatory status that may be relevant, as discussed intensively during this workshop. Recent reports have shown that signs of inflammation such as increased CRP or IL-6 are independent of the IRP and must be accounted for separately. In the National Health and Examination Survey (NHANES) III study, combined CMV-seropositivity and higher CRP levels were associated with increased mortality. A relationship between functional ability in older people and the immune system has been documented in several studies: frail elderly individuals have higher CMV titers, elevated IL-6 and lower responses to influenza vaccination, but the interrelations between these are not clear. It is also not clear if CMV reactivates more often in the elderly with or without frailty and if higher levels of antibodies against CMV are associated with lower survival.

Studies of CMV prevalence in different populations have shown high variability. In elderly Chinese Singaporeans, CMV prevalence is very high (99%) as shown by **Larbi** (Singapore). Since 2003, the Singapore Longitudinal Ageing Study (SLAS) recruiting individuals over 55 years of age has characterized over 4000 study participants for nutritional, behavioural, metabolic, social, and biological parameters. The extensive clinical information collected over the years enables us to now identify immune parameters associated with chronic conditions (eg. diabetes, hypertension, high cholesterol). A global approach including immune monitoring and bioinformatics should enable the identification of immune correlates of longevity and co-morbidities, in a systems biology approach. Data presented highlighted that immunological history in Asian elderly (eg. Dengue, H. pylori, CMV, EBV) is different from in other parts of the world. This may be of major importance for the identification of other or additional driving forces than CMV leading to immunosenescence. CMV IgG levels are different in CMV-seropositive elderly depending on the presence of co-morbidities (eg. diabetes) suggesting that stratification of elderly individuals based on any parameter related to chronic conditions should be considered. Correlations between CMV IgG and inflammatory markers were presented and also shown to correlate with the frequency of differentiated CD8+CD28-CD27- T cells. The data presented suggest that better stratification of the elderly should be performed in order to understand the role CMV in healthy ageing and how other conditions may synergize or conflict with CMV-induced effects at the immunological level.

Derhovanessian (Tübingen, Germany) presented data on familial longevity as illustrated by the Leiden Longevity study (LLS), which includes 450 families in which offspring enjoy a standardized mortality rate 30% lower than their partners from the general population. Offspring from long-lived families had a significantly lower number of late-stage, possibly terminally, differentiated CD8+ T cells (CD45RA−CCR7−CD27−CD28−) and latent CMV infection did not have the same impact on the percentage of naive (CD45RA+CCR7+CD27+CD28+) and late-differentiated effector memory CD8+ T-cells as it has in the rest of the population. Thus, the decrease in naïve and accumulation of late-differentiated “senescent” CD8+ T cells that is commonly taken as a hallmark of immunosenescence was not seen in these subjects. CMV-associated pro-inflammatory status (assayed as CRP levels) in CMV-seropositive offspring from long-lived families was also lower than in the general population, but no differences in the cellular responses to CMV *in vitro* were found. However, analysis of the serological response to CM2, a fusion protein containing the C-terminal portion of viral protein pUL44 and a highly reactive fragment of pUL57, associated with active infection, revealed that the percentage of individuals with detectable levels of IgM and IgG antibodies to CM2 was lower in the offspring compared to their partners; in parallel, a lower percentage of naïve CD8+ cells and higher percentage of late-differentiated CD8+ cells was seen in subjects with CM2-binding IgG and IgM antibodies. No differences were found in naïve CD4 cells. These data suggest either a lower reactivation rate of CMV in offspring predisposed for familial longevity, or better immune control of the virus on reactivation, and may help to explain the absence of CMV-associated markers of immunosenescence in these individuals [3].

The identification of phenotypes that could be used to anticipate which individuals are at higher risk for immunosenescence and mortality (“biomarkers”) remains elusive. The analysis of 2 year survival in a cohort of donors over 65 from the south of Spain (Sevilla, Spain) confirmed that CD4/CD8 ratios below 1 as well as markers of inflammation (neutrophilia, high CRP levels, IL-6) and thymic function (indirectly calculated in peripheral PBMC DNA using the sj/β-TREC ratio, [4]), was associated with increased risk of death from any cause (Ferrando-Martinez, Seville, Spain). Multivariate analysis showed that lower thymic function, higher CRP levels, and presence of neutrophilia were independently associated with time to death in this cohort. Based on these results the use of the “CRT” index (CRP and Thymic function) was proposed to define a biomarker profile to identify individuals at higher risk of death [5]. There were some limitations to this study regarding the role of CMV because most of the elderly in Spain are CMV-seropositive, as in Singapore. We therefore need a more sophisticated analysis than mere sero-positivity or –negativity, because it is clear that the way that an individual deals with the infection is very important (as illustrated in the LLS by Derhovanessian, mentioned above). This question was approached by Hurme (Tampere, Finland) who presented results on transcriptomic analysis of CMV reactivation in seropositive nonagenarians. The presence of CMV DNA was found not to correlate with T cell subset distribution or with the levels of inflammatory markers. In the Vitality study, a cohort of 131 nonagenarians, a genome-wide gene expression array was performed. The results showed that 55 genes were upregulated and 65 genes downregulated. The most highly upregulated gene is the H3F3C gene in the PKA pathway. It is questioned whether the presence of CMV DNA is due to passive release, rather than due to an active, productive infection and the possibility of an inefficient immune elimination.

### Is immunosenescence treatable

Cicin-Sain (Braunschweig, Germany) and Nikolich-Zugich (Tucson, Arizona, USA) analyzed cause-effect relationships between CMV infection and age-associated changes in the immune system using an experimental murine model of life-long CMV infection. Several changes consistent with the development of an IRP (e.g. enrichment of effector memory CD8 cells) were observed in mice infected with MCMV but not in control mice infected with non-persistent virus or with other herpesviruses. MCMV infection resulted in loss of CD8 T-cell functional activity against other viruses that correlated with the accumulation of MCMV-specific EM cells, suggesting that these cells may compete with the responses against novel antigens [6,7]. In general, therefore, these results in mice are similar to those seen in humans. It may therefore be appropriate to use this animal model to investigate the effects of anti-viral agents on immunosenescence in the elderly.

To this end, Beswick and Moss (Birmingham, UK) presented a model using elderly mice with latent CMV infection to analyze whether antiviral therapy can reverse the development of immune senescence. Administering valaciclovir antiviral treatment for 12 months significantly reduced the frequency of MCMV-specific T-cells in 18 month-old mice with pre-existing memory inflation and the residual immune response was less highly differentiated. Furthermore, this treatment lead to a restoration of the frequency of naive CD8+ T cells, and improved the *de novo* immune response to Influenza challenge as seen by improved survival and a higher frequency of Influenza- specific lymphocytes in the mediastinal lymph nodes. In addition, MCMV infection with the attenuated *tsm5* virus (mutated DNA-polymerase) did not elicit memory inflation and therefore these investigators concluded that lytic viral reactivation is key to the accumulating MCMV-specific immune response. If these results could be translated into clinical practise, the implications would be very exciting.

Nikolich-Zugich (Tucson, Arizona, USA) also presented data on CMV infection and T cell ageing in mice showing that lifelong CMV infection leads to an increased mortality in aged mice, reduced immune response to infections and reduced polyfunctionality of lymphocytes. In humans, a search for the IRP and the effect of CMV in a cross-sectional analysis of a mixed US cohort showed that the absolute loss of naïve cells was due to ageing as it occurred in both CMV+ and CMV- individuals, whereas the increase of CD8+ EM cells and a decrease of the total CD8+ T cell pool were due to CMV infection. There is some controversy as to the effect of CMV in this context, because some studies have shown that a significant age-associated deficit of naïve CD8+ T cells is not seen in CMV-seronegatives, or at least not to anything like the same extent. A more detailed consideration of the impact of CMV on these parameters is therefore required.

### The effect of ageing and CMV on adaptive immunity

A diverse T cell repertoire is required to develop effective pathogen-specific immunity, and it is therefore important to assess virus-specific T cells and those recognizing other pathogens in elderly people. Wang (Baltimore, Maryland, USA) presented a study on CMV-specific repertoire diversity and antibody levels in young and older adults. Using a single-cell strategy for clonotypic analysis of the TCRαβ repertoire of CD8+ T cells, they analyzed the diversity and magnitude of the CMV-specific CD8+ T-cell response. It was found that TCRαβ diversity, but not the size of the T-cell response, was inversely related to antibody levels against CMV, which in turn was associated with the detectability of circulating viral DNA. These results indicate that the CMV-specific CD8+ TCRαβ repertoire diversity may be more important than the size of the CD8+ T cell response in viral control [8].

A major question remains whether age impacts on the breadth, frequency and stability of CD4 and CD8 T cell specificities in healthy donors, and to what extent CMV is responsible for any such effects? Wills (Cambridge, UK) tried to answer that question by analyzing the response towards multiple CMV antigens. It was found that IE-1-, pp65-, US3- and pp71-specific CD8+ T cells are able to control the dissemination of virus *in vitro*. The ability of CMV-specific CD8+ T cells to control virus dissemination was not affected by donor age. Young, middle aged and old individuals had good CTL responses and produced IFN-γ. CD4 cells target the latent proteins UL138 and LUNA; a proportion of this response is mediated by Th1 cells while other cells involved in of the response secrete the immunosuppressive cytokines IL-10 and TGF-β. These results emphasize the importance of maintaining effective immunosurveillance against CMV at any age. This work challenges the opinion that the immune response to CMV in the elderly is focused on a small number of proteins and is characterized by lower functionality compared to younger individuals. **Kern** (Brighton, UK) presented an analysis of T cell response to 19 different CMV targets previously identified to be the most relevant CD4 and CD8 T-cell target antigens in the CMV proteome [9]. Using multi-parameter flow cytometry and intracellular cytokine staining (ICS) they evaluated multiple functions and phenotype markers in parallel, including IL-2, TNF-α, IFN-γ, CD40L, degranulation, and the memory surface markers, CD45RA *and* CD27. No significant age-associated differences with respect to CD4+ T cell responses were found in their preliminary data analysis, but there was an apparent increase in the size of the CD8+ T cell response in older age (manuscript in preparation). Those individuals with the greatest single protein-specific responses had retained broad responses in terms of target recognition. In addition, there was no apparent reduction of functional breadth, neither in CD4+ nor CD8+ T-cells. This seems to indicate that responses do not concentrate on fewer specific target proteins as a consequence of aging*.* These results seem to support the notion that neither the quantity nor the quality of the T cell mediated anti-CMV response is affected by ageing. Reports published several years ago seemed to indicate that with respect to very select specificities, there may be a functional loss [10]. This is likely owed to differences in technology, where staining of CD8 T-cell with tetramers detects non-functional cells that will not be detected using ICS. However, the results from Kern’s lab clearly show that CMV-specific polyfunctional T-cells do not decrease in absolute numbers with advancing age, suggesting that non-functional T-cells might occur in addition to but not in the place of fully functional T-cells [11]. Riddell and Akbar (London, UK) analyzed the avidity of CMV-specific CD8 T cells in the elderly. CMV infection induces the accumulation of CMV-specific CD45RA+ memory CD8+ T cells. Tetramer binding avidity correlates inversely with CD45RA expression whereas high TCR avidity is associated with enhanced effector function (CD107 expression, IFN-γ and TNF-α production). The functional characterization of HLA-A*0201-restricted pp65-specific CD8+ T cells that accumulate with age showed that they had low avidity. The causes of this accumulation and implications for immunosenescence are open questions.

There is limited quantitative data on clonal diversity and its evolution over time after primary infection, or on cell phenotypes in immune tissues other than blood. Van Lier (Amsterdam, The Netherlands) presented data on TCR diversity in healthy donors and kidney transplant recipients using high throughput sequencing of CDR 3 regions. The clonal distribution of CMV-specific clones is relatively stable. IE-1 responses differ in that they are more restricted. CMV-specific cells represented a minor fraction of the CD8+ T cell pool found in the lymph nodes. In contrast to peripheral blood, pp65-reactive CD8+ T cells from lymph nodes resemble central memory-like cells [12]. It is suggested that CMV-specific clones in lymph nodes can be recruited into the circulating pool upon CMV reactivation.

Sansoni (Parma, Italy) explored anti-CMV CD8+ T cell responses in a cohort of CMV- seropositive elderly individuals. The results indicate that absolute numbers of anti-CMV CD8+ T cells did not significantly change with age. Increasing age correlates with the loss of naïve CD8+ T cells, but not with the expansion of CD28+ memory or CD28− effector CD8+ T cells. By contrast, the magnitude of anti-CMV responses is not correlated with naïve CD8+ T cells, but strongly correlates with the accumulation of antigen-experienced CD8+ T cells. These results suggest that there is a dichotomy between age and anti-CMV responses acting as independent factors subverting the naïve pool and EM cell subset respectively.

The accumulation of large numbers of CD8+ effector T cells, frequently CMV-specific, may hamper the effect of vaccination and exacerbate the development of age-related diseases. Grubeck-Loebenstein (Innsbruck, Austria) reported that resting CD8+ CD28− effector T cells, that are more prone to undergo apoptosis following DNA damage, can be rescued by cytokines. Thus, CMV-specific CD8+ CD28− T cells may survive and accumulate in the bone marrow, specifically in the elderly, due to high IL-15 and IL-6 production. It is suggested that in elderly individuals, CD8+ CD28− T cells may represent a useful line of defence against pathogens such as CMV and may compensate for the loss of naïve and early memory T cells. In another set of experiments aimed at analyzing *in vitro* the production of cytokines by fibroblasts in young and elderly individuals it was observed that CMV infection induced the production of IL-6 and IL-8, particularly in old age. Thus, lifelong infection with CMV may contribute to age-related inflammatory processes, referred to as inflammageing. Clearly, CMV infection affects many cell types and has wide-ranging direct and indirect effects also on innate immune mechanisms [13,14].

### HCMV infection, ageing and innate immunity

Lopez-Botet (Barcelona, Spain) analyzed NK cell responses to CMV. CMV- seropositivity is associated with a variable and persistent increase of NKG2C^bright^ NK cells in healthy adults and children. NKG2C^bright^ NK cells do not co-express NKG2A, display lower levels of NKp30 and NKp46 activating receptors and include higher numbers of cells bearing LILRB1 and KIR inhibitory receptors specific for HLA class I molecules. The proportions of NKG2C+ NK cells and the NKG2C^bright^ phenotypic profile appeared comparable in HCMV+ individuals of different ages (median: 19, 49 and 70 years). CMV infection has been reported to induce a marked NKG2C^bright^ differentiation and expansion in kidney and stem cell transplantation recipients and in immunodeficient patients. It is hypothesized that the interaction of the activating CD94/NKG2C receptor with a ligand on infected cells, together with cytokine-mediated signaling (e.g. IL-15), may induce the differentiation and expansion of this NK cell subset.

Several age-related changes in NK cells were reported by Solana (Córdoba, Spain). In elderly individuals, the percentage of NK cells is increased. The analysis of NK cell subsets in the elderly shows an increase of the more mature CD56^dim^ NK cells and a decrease of CD56^bright^ NK cells. The expression of CD16 allows the identification of a novel CD56−CD16+ subset that is increased in the elderly. Analysis of activating receptors shows that NKp30, NKp46 and DNAM-1 are significantly decreased in the elderly. Decreased per-cell NK cell cytotoxicity and a decreased capacity of NK to collaborate in DC maturation (NK-DC crosstalk) are observed in elderly individuals. The remodeling of NK cell subsets together with the decreased expression of NCRs and DNAM-1 may contribute to explaining the functional alterations found in NK cells from the elderly [15,16].

A key component of the innate immune system is mannose-binding lectin (MBL). Naumova (Sofia, Bulgaria) studied gene polymorphisms that have been described to correlate with MBL production capacity [17]. The low secretor haplotypes are very common worldwide, making MBL-deficiency the most common form of immune deficiency. MBL deficiency is frequently asymptomatic, but may become symptomatic when associated with other immune system stressors. The relevance of two innate immunity gene systems KIR and MBL2 for successful ageing was discussed. MBL2-deficient haplotypes are associated with high levels of anti-CMV antibodies in the elderly.

### Extrinsic factors affecting immunosenescence: role of CMV?

Bosch (Amsterdam, The Netherlands) analyzed the effects of CMV on immunity in young adults, and the role of life style and psychological stress on the immune response to CMV. The analysis of a cohort of 160 healthy university students showed that CMV-seropositive individuals had a reduction in their CD4:CD8 ratio, an increase of EMRA T cells, elevated plasma IL-6 and lower response to influenza vaccination. These data suggested that rudimentary signs of immune senescence can already be observed in CMV-infected young adults. In another cohort study (Augsburg EADS, 950 factory workers, mostly males) an association of low income and low education status with CMV-seropositivity was observed. Moreover, in CMV-seropositives, the increase of late differentiated CD8 T cells was positively correlated with lower income and lower education status.

Rector, (Birmingham, UK), analyzing the same cohort, found that smokers were 79% more likely to be CMV+. Other lifestyle factors, such as alcohol consumption and exercise, were not significant. Cardiovascular risk factors like triglycerides and several psychological factors such as sleep disturbances, vital exhaustion, depression, and self-assessed mental health were positively correlated with CMV titers. These findings raise the possibility that the associations between morbidity/mortality and CMV observed in older adults may be secondary to life style and socio-economic correlates of CMV infection in young and middle-aged adults, setting the stage for poorer health in later life. The analysis of differentiated EM and EMRA CD8β cells showed that, this population decreased with increasing age in those that were CMV+, but not in those that were CMV–.

### Clinical implications of latent HCMV infection and immunosenescence

The impact of measurement errors on the relationship between CMV infection and clinical outcome was explored by Wang (Baltimore, Maryland, USA) by a strategy of data simulation based on data and regression coefficients from the Women’s Health and Ageing Studies. The results from this study showed that measurement errors lead to a significant underestimation of the effect of CMV infection on chronic disease and mortality risk that should be considered in these studies.

It is well-known that elderly individuals generally have a worse response to vaccination than the young. Govind (Cranfield, UK) presented data from the MARKAGE European Study to establish biomarkers of human ageing. In order to identify those individuals who will not respond effectively to vaccination, the group aims to identify a set of biomarkers of ageing. Absolute quantification of herpes viral load achievable in urine (CMV, HHV6a and 6b are detectable) may represent a useful non-invasive diagnostic method for immune competence in older individuals.

McElhaney (Vancouver, Canada) presented data on biomarkers of “inflammageing”, the chronic elevation of inflammatory mediators that weakens the immune system as we age. A key role in inflammageing may be played by chronic CMV infection that stimulates and thereby exhausts the immune system, as previously mentioned. In response to influenza vaccination, a high proportion of potentially senescent CD8+ T cells do not co-express Granzyme B (GrzB) and Perforin. Using the baseline level of GrzB, a biomarker called bGrzB has been developed. The level of bGrzB increases with age and is higher in CMV+ than in CMV− donors. GrzB is co-localized with CD8+ T cells in atherosclerotic lesions leading to plaque instability. In heart failure, GrzB is released from CD8+ T cells most probably due to the chronic inflammatory stimulus associated with this disease.

Frasca (Miami, Florida, USA) assessed activation-induced cytidine deaminase (AID) as a biomarker of B cell function. AID is crucial for somatic hypermutation and class-switch recombination*.* A reduced serum response of the elderly to influenza vaccination assessed by ELISA and hemagglutination inhibition assays is commonly observed. *In vitro* AID responses to CpG were also decreased with age and correlated with serum response. The age-associated defects in B cell function may be due to the increased levels of systemic TNF-α (which are positively correlated with levels of CMV IgG), which induce more TNF-α production by B cells and this pre-activated status of the B cells renders them refractory to undergo *in vitro* class switching.

The NHANES III study on nutrition and health in the USA between 1988 and 1994 was a cross-sectional, multistage, stratified, clustered probability sample of the US civilian non-institutionalized population that included CMV serology. This information was used by Hill (Portland, Oregon, USA) to test the association between CMV seropositivity and mortality, using Cox logistic regression. Consistent with the results of Aiello mentioned below who had previously analyzed the same dataset, it was found that CMV produces a modest increased risk for all-cause mortality. The impact of CMV was largely explained by an increase in cardiovascular (CVD) deaths. CMV’s main impact was seen in individuals aged 55–75 at the time of the survey, and particularly in the 55–65 year age group. CMV imposed little increased risk of either all-cause or CVD mortality in the most elderly (aged 75–90). High cholesterol was associated with CVD mortality only in the younger age group. These results may reflect a selective loss from the population of individuals with the highest risk of CVD mortality due to unknown or unmeasured factors.

Aiello (Ann Arbor, Michigan, USA) presented data on CMV, stress and immune markers of ageing. The social gradient in health may act in part through stress pathways. Reactivation of herpesviruses is considered a hallmark of psychosocial stress. Using data from NHANES III, Aiello and colleagues found that those with lower income and education levels were more likely to be CMV seropositive and have higher IgG antibody titers against CMV than those with higher income and education [18]. Social patterning of infection and immune response against CMV may reflect increased likelihood of exposure, higher dose of infection and/or poorer immunological control of CMV. CMV seropositivity as well as elevated CMV IgG antibody titer have, in turn, been associated with increased risk for all-cause mortality [19,20]. Using data on persons 18 years of age and older from the Detroit Neighborhood Health Study, Aiello et al. examined the possible immunological mechanisms which may link CMV to mortality. Elevated CMV IgG antibody titer was associated with an increased ratio of late differentiation-stage EMRA T cells to naïve cells even after controlling for age, medication use and co-infection with herpes simplex virus-1. Therefore, immunological ageing due to elevated immune response against CMV may be a novel biologic pathway by which psychosocial stressors impact risk for mortality.

Persistent infection with CMV is thought to be a key factor for the amplification of the inflammation associated with ageing. Maier (Leiden, The Netherlands) presented an analysis of the innate immune capacity, measured by LPS induced cytokine production capacity of whole blood, and CMV infection in three different cohorts, the Leiden Longevity Study, Prosper Study and Leiden 85-Plus Study. However, levels of proinflammatory and anti-inflammatory cytokines produced upon LPS stimulation showed no correlation with CMV serostatus or with CMV IgG levels in these three cohorts. However, cytokine production capacity from CMV– or CMV+ donors showed high interindividual variability and reflected survival propensities and metabolic disease. Analyses on comorbidities and CMV infection showed higher prevalence of diabetes, higher non-fasting glucose levels and glycosylated haemoglobin (HbA1c) in CMV+ oldest old participants, but there was no correlation with CMV IgG titers [21].

The possible relationship between diabetes mellitus (DM) Type 2, elderly, frailty and CMV have been analyzed by Fülop (Sherbrooke, Canada). CMV-seropositivity is more prevalent in diabetic elderly subjects. It seems that chronic disease is a more important determinant for frailty than the CMV status. CMV+ elderly DM patients have less putatively senescent cells than healthy CMV-seropositives but express more CD57 at the single cell level which is more accentuated in CD8+ T cells. After influenza vaccination, the highest level of GrzB is found in CMV− DM patients that had the lowest level of potentially senescent cells. Diabetes seems to suppress the percentage of these CD8+ T cells.

The possibility that infection by CMV or other herpesviruses could be related to inflammatory diseases has been explored by Sauce (Paris, France) who analyzed the capacity to respond to CMV and EBV by Systemic Lupus Erythematosus (SLE) active and inactive patients. A discrepancy between CMV and EBV response was observed in SLE patients. Whereas CMV responsiveness was not altered, the response to EBV was depressed, with lower IFN-γ, TNF-α, MIP1β and IL-2 secretion and lower cytotoxicity. This dysfunctionality can be due to an exhausted T cell phenotype (PD-1^hi^) since PD-1 blocking restores responsiveness [22].

CMV is still regarded as being the most significant infectious pathogen in the solid organ transplant recipient, and in spite of the improvements in surveillance and treatment, it continues to be a major cause of morbidity and mortality after transplantation and is associated with lower graft survival. Appay (Paris, France) focused on the role of CMV in the pathogenesis of acute rejection in lung transplantation. An association between cellular immune activation (i.e. expression of CD38 on T cells) and acute rejection was found. Levels of total CD38+ CD8+ T cells correlated with the frequency of CMV-specific cells. The development of pro-inflammatory CMV-specific CD8+ T cell immune responses may explain the relationship between CMV infection and acute lung rejection. It is suggested that potent CMV prophylaxis should be given to all CMV-seropositive patients to prevent the occurrence of acute rejection. CD8+ T cell activation levels in peripheral blood correlate well with CD8 responses in the lung and may predict the risk of acute rejection. Zielinski (Gdansk, Poland) analyzed CMV infection in renal transplantation patients. In a retrospective study, it was observed that elderly kidney recipients, CMV-positive with high numbers of CD28− T cells and short telomeres, had fewer episodes of acute rejection. Preliminary data suggest that CMV challenge has a strong impact on the immune system after allotransplantation in the elderly. Solana and Cantisan (Cordoba, Spain) reported an analysis of CD45RA+ (EMRA) CMV-specific CD8 T cells in relation to CMV replication parameters in solid organ transplantation. It was observed that EMRA increase by 4% for each log of viral replication. Replication is associated with continuous and constant increases in percentages of CD28− cells. The impact of CMV on inducing expansion of CD28− CMV-specific CD8 T cells is seen mainly in young individuals [23].

### Future directions and concluding remarks

Significant advances in the understanding of the changes of immune system associated with CMV infection and their possible significance for immunosenescence have been made over the past few years in many areas of medicine, as reported here.

In this workshop, many important questions have been addressed and other new questions been raised. A panel of markers to assess T cell phenotypes in T cells subsets in studies of immunosenescence and CMV infection was proposed and discussed, as well as the significance of the IRP phenotype, markers of thymic function or inflammation on survival in the elderly. The role of CMV in immunosenescence has been confirmed in experimental models, opening new perspectives to explore possible therapies aimed at reversing immunosenescence. However, a major question still remains concerning the mechanisms driving the homeostatic fluctuations of CD8 T cells during latency of CMV infection, and how age impacts on the breadth, frequency, phenotype and function of CD4 and CD8 T cell specificities in healthy donors. Although primarily affecting the T cell compartment, evidence is accumulating in support of a significant effect of CMV infection and ageing on innate immunity as well, in particular NK cells.

**Figure 2:**
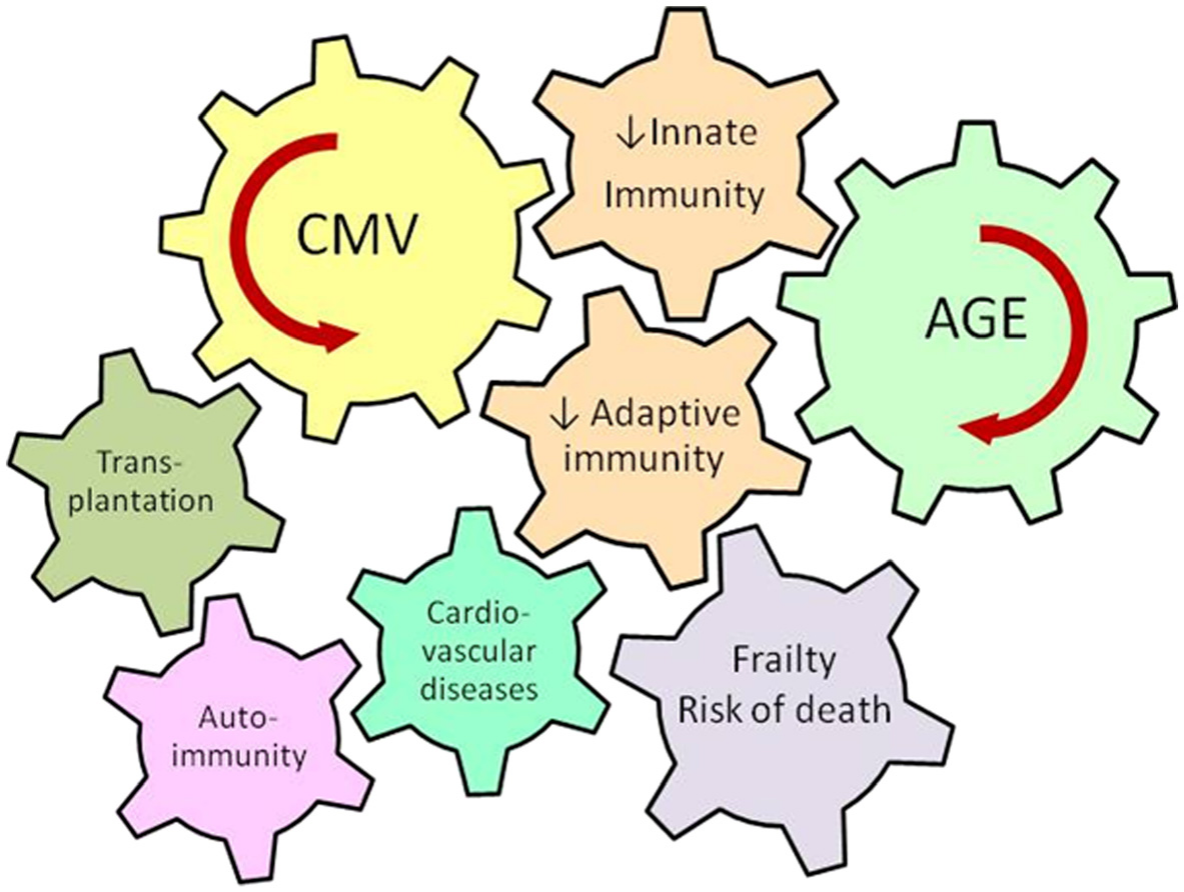
**Age and CMV infection are major driving forces contributing to the deterioration of innate and adaptive immunity.** Age-associated decrease of adaptive immunity is termed immunosenescence. The deregulation of innate immunity is associated with inflammageing. Immunosenescence and inflammageing play a significant role in the pathogenesis of different clinical situations that can lead to increased risk of frailty and death in the elderly.

Results from translational research of CMV infection and immunosenescence in clinical conditions such as transplantation, cancer, immunodeficiency and autoimmune and inflammatory diseases, supports the notion that CMV can affect their evolution and prognosis by inducing a process of “early” immunosenescence (Figure 2). Furthermore, other extrinsic factors can also contribute, together with CMV, to the age-associated deterioration of the immune system.

However, there are still many open questions about the immune response to HCMV itself in ageing and about the role of CMV in early immunosenescence (Figure 1):

a) It is likely that humans can be infected with multiple CMV strains. How does this affect their interactions within the immune system and their effect on immunosenescence?

b) What is the real impact of the complexity of CMV infections on the processes of T cell homeostasis and differentiation in the course of ageing?

c) It is necessary to test the function of T cells in ageing and CMV infection before they are labeled as “senescent”, “exhausted” or in other manner inadequate.

d) What is the contribution of CMV- induced inflammation to inflammageing? What are the best markers that could be used to define CMV-induced systemic inflammation?

It was proposed that the organization of a 4^th^ workshop is vital for the field to move forwards, to answer these and other questions. We are grateful to Paolo Sansoni who agreed to organize it in Parma (Italy) in 2013.

## Competing interests

The authors declare that they have no competing interests.

## Authors’ contributions

All authors attended the Workshop, participated in the discussion, saw and commented on the text published here. All authors read and approved the final manuscript.
